# Exploring the link between dietary thiamine and type 2 diabetes mellitus risk in US adults aged 45 years and older: Insights from a cross-sectional investigation

**DOI:** 10.1371/journal.pone.0313114

**Published:** 2024-12-06

**Authors:** Hong Lin, Zhengwei Gao, Hengfan Ni, Jian Li, Haoran Liu, Bo Qin, Zhiyao He, Zhaohui Jin

**Affiliations:** 1 Department of Pharmacy, West China Hospital, Sichuan University, Chengdu, Sichuan, China; 2 Department of Pharmacy, Sichuan Provincial Maternity and Child Health Care Hospital, Affiliated Women’s and Children’s Hospital of Chengdu Medical College, Chengdu, Sichuan, China; Bowen University, NIGERIA

## Abstract

The correlation between dietary thiamine intake and the incidence of type 2 diabetes mellitus (T2DM) remains a subject of controversy within the academic community. While numerous studies have attempted to elucidate this relationship, conclusive evidence remains elusive. A survey of U.S. adults aged 45 years and older examined the supposed association between dietary thiamine intake and the risk of developing T2DM with the aim of clarifying the potential link. In this cross-sectional investigation, we evaluated dietary thiamine intake data sourced from the National Health and Nutrition Examination Survey (NHANES) from 2007 to 2020. Using weighted multivariate logistic regression analysis, we assessed the potential risk of T2DM associated with varying levels of thiamine intake. The observation of nonlinear relationships was accomplished by fitting smoothed curves. This study ultimately included 15,231 participants aged 45 years and older. Dietary thiamine intake (after log transformation) was inversely related to T2DM after accounting for potential confounders (OR = 0.86, 95% CI: 0.78, 0.95). An increase in dietary thiamine intake by one unit is associated with a 14% reduction in the risk of T2DM. Furthermore, our analysis revealed that the associations between dietary thiamine intake and T2DM risk, such as age, gender, race, smoking status, alcohol use, hypertension, body mass index (BMI), and cardiovascular disease (CVD), remained consistent across multiple stratified subgroups (p values >0.05). According to this study, dietary thiamine intake may be associated with the incidence of T2DM among US residents aged 45 years and older. Appropriate increases in dietary thiamine intake are expected to offer substantial preventive potential for T2DM and significant clinical implications.

## Introduction

Insulin resistance and compromised glucose metabolism are pivotal characteristics of type 2 diabetes mellitus (T2DM). The incidence of T2DM has increased significantly over recent decades, becoming a significant global health concern [1,2]. It is projected that by 2045, the global population of individuals diagnosed with diabetes could increase to 780 million, with over 90% of these cases attributed to T2DM [3]. The profound increase in T2DM cases worldwide underscores the urgent need for further examination and effective intervention strategies to address this mounting epidemic. T2DM complications such as atherosclerosis, stroke, diabetic retinopathy, and end-stage renal disease can lead to a severe financial burden [4,5]. The dramatic increase in T2DM patients can be attributed to an aging population, economic development, urbanization, dietary habits, and a sedentary lifestyle [6]. In particular, dietary factors have received considerable attention from researchers [7–10]. This includes micronutrients that have received some attention for their impact on T2DM risk.

Thiamine is an important cofactor necessary for metabolic reactions involving glucose, mainly as thiamine pyrophosphate [11]. As part of pentose phosphate metabolism, thiamine is used to synthesize neurotransmitters, nucleic acids, fatty acids, amino acids, and steroids. It is also an important cofactor in the production of energy in the tricarboxylic acid cycle [12]. Thiamine, an essential vitamin, can be sourced from a diverse range of foods, including pork, fish, nuts, asparagus, and whole grains, among others [13]. The presence of thiamine in these food items contributes to their nutritional value. However, despite the widespread availability of thiamine in food, micronutrient deficiencies are still prevalent globally. Thiamine deficiency has been reported to lead to neurological disorders such as Beriberi, Wernicke’s encephalopathy, and migraine [14–16].

Recent studies indicate that dietary thiamine may be associated with T2DM. This may explain part of the favorable relationship, but the conclusions of these studies remain controversial. Epidemiological studies have revealed a negative correlation between thiamine intake and the incidence of T2DM, suggesting that thiamine may have a potential protective effect on the development of this chronic disease [17]. Additionally, an analogous trend has been noted in individuals diagnosed with gestational diabetes mellitus (GDM) [18]. Furthermore, a double-blind trial demonstrated that high-dose thiamine supplementation effectively lowered fasting blood glucose levels in hyperglycemic patients, halted the progression of insulin dysfunction, and significantly enhanced glucose tolerance [19]. Nonetheless, conflicting viewpoints exist in the literature regarding this matter [20,21]. Drawing from the aforementioned association, we hypothesize that there is a potential correlation between dietary thiamine intake and the likelihood of developing T2DM. The hypothesis was tested using a representative sample of the American populace drawn from National Health and Nutrition Examination Survey (NHANES) data.

## Materials and methods

### Study population

**Fig 1:**
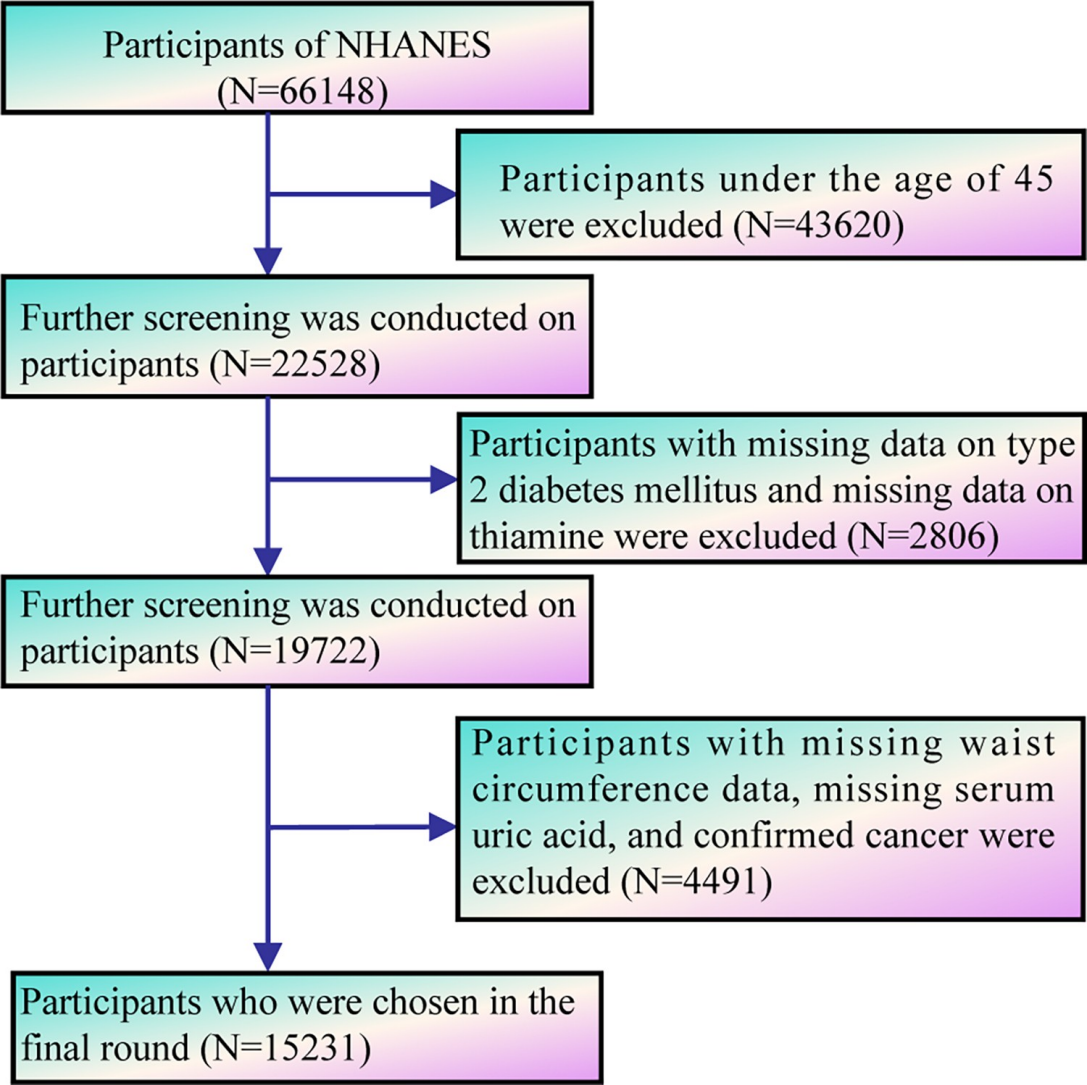
Population flow chart for the study.

This study utilized data from the NHANES spanning from 2007 to 2020, employing a nationally representative sample to ensure comprehensive and reliable analysis. The dataset, along with detailed descriptions of the data collection procedures and laboratory examination methodologies, is publicly accessible and freely available on the NHANES website (https://wwwn.cdc.gov/nchs/nhanes/). According to the National Center for Health Statistics (https://www.cdc.gov/nchs/nhanes/irba98.htm), the NHANES has been approved by the NCHS ethics review board. In addition, in accordance with rigorous ethical standards, all survey participants were asked to provide written informed consent. In all, 66,148 people were a part of this research. Following screening, the following individuals were deemed ineligible: 43,620 were under the age of 45; 2,806 had missing data for T2DM and thiamine; and 4,491 had missing data for waist circumference, serum uric acid, or cancer diagnosis. After careful consideration, the analysis was expanded to include 15,231 persons (aged 45 and up) who fulfilled the inclusion criteria (Fig 1).

### Exposure variables

Study participants were prompted to record their food and beverage intake across two consecutive 24-h periods (spanning from midnight to midnight) for the purpose of calculating their thiamine dietary intake. The initial dietary recall test was administered at the Mobile Examination Center (MEC). Subsequently, a telephone follow-up was conducted within 3 to 10 days. For participants who provided two 24-h recalls, the average was computed; otherwise, a single 24-hour recall was utilized.

### Outcome variables

This study examined T2DM as the outcome variable. NHANES participants who had diabetes were diagnosed according to US diagnostic criteria or self-reported diabetes [22,23]. T2DM was self-reported, glycosylated hemoglobin was at least 6.5%, fasting blood sugar level was at least 126 mg/dL, and antidiabetic medications were taken daily.

### Covariates

These confounding factors were selected based on previous research and experience [17,20,21,24]. Basic characteristics of the population: age, gender, race, education, and poverty income ratio. Lifestyle: Smoking status, alcohol use, and physical activity. Health checkup: Body mass index (BMI) and waist circumference (WC). Laboratories: Total calcium, blood urea nitrogen (BUN), total bilirubin (TBIL), serum uric acid (SUA), triglyceride (TG), total cholesterol (TC), high-density lipoprotein cholesterol (HDL-C), estimated glomerular filtration rate (eGFR), and the urine albumin-to-creatinine ratio (UACR). Disease diagnosis: hypertension and cardiovascular disease (CVD). Hypertension was defined as a systolic blood pressure (SBP) ≥130 mmHg or diastolic blood pressure (DBP) ≥80 mmHg, diagnosed by a physician or prescription medication [25]. CVDs were identified through self-reports and included congestive heart failure (CHF), coronary heart disease (CHD), angina, heart attack, and stroke.

### Statistical analyses

In this study, we utilized R version 4.2.0 in conjunction with the EmpowerStats software package to perform our analyses. The data were accessed on April 25, 2024, from https://wwwn.cdc.gov/nchs/nhanes/. We adhered strictly to the Centers for Disease Control and Prevention (CDC) guidelines for weights in our calculations. Given the skewed distribution of dietary thiamine intake, we applied a log2 transformation to normalize the data and subsequently categorized them into quartiles (labeled Q1, Q2, Q3, and Q4). We computed the mean (95% CI) for continuous variables and determined p values using either the Kruskal‒Wallis test or weighted linear regression models. Categorical variables were analyzed using weighted percentages (95% CIs), and p values were calculated through weighted chi-square tests. To investigate the relationship between dietary thiamine intake and T2DM, we followed the Strengthening the Reporting of Observational Studies in Epidemiology (STROBE) guidelines [26,27]. We conducted weighted univariate and multivariate logistic regressions to thoroughly evaluate this correlation. Our analysis included three models: Model 1 was unadjusted; Model 2 was adjusted for age, gender, and race; and Model 3 was further adjusted by incorporating additional covariates. For robustness, we grouped dietary thiamine intake into quartiles, using their median values for trend analysis as a continuous variable. Additionally, smoothed curve fits were plotted to ascertain whether a nonlinear relationship existed between dietary thiamine and T2DM. A likelihood ratio test was conducted to evaluate potential interactions among subgroups, such as age, gender, race, smoking status, alcohol use, BMI, and hypertension. Forest plots were generated to visualize these interactions. Statistical significance was determined at a p value less than 0.05.

## Results

### Statistical characteristics of the population

We characterized the population of participants according to whether they had T2DM (Table 1). This sample had a mean age of 59.1 (58.8, 59.4) years, 47.9% were male, and 71.7% were non-Hispanic white. In the analysis of whether participants had T2DM, age, gender, race, education, poverty income ratio, alcohol use, smoking status, thiamine intake, physical activity, BMI, WC, total calcium, BUN, TBIL, SUA, TG, TC, HDL-C, UACR, hypertension, CHF, CHD, angina, stroke, heart attack, and CVD were significantly different between the groups (p<0.05). To further observe the distributional characteristics of the population in terms of dietary thiamine intake. We classified participants into quartiles of dietary thiamine intake: Q1, Q2, Q3, and Q4 (Table 2). A total of 19.8% of the patients had T2DM. In the quartile analysis of dietary thiamine intake, age, gender, race, education, poverty income ratio, alcohol use, smoking status, physical activity, BUN, total calcium, TBIL, SUA, TG, TC, HDL-C, eGFR, WC, hypertension, CHF, stroke, and T2DM were significantly different between the groups (p<0.05). In those indicators where there was a difference, the following decreased with increased dietary thiamine intake: age, females, non-Hispanic Blacks, other races, those with education lower than high school, poverty income ratio below 1.3, poverty income ratio between 1.3–3.5, never alcohol use, vigorous activity, TC, HDL-C, CVD, CHF, stroke, and T2DM. In contrast, the following factors increased with increased dietary thiamine intake: males, non-Hispanic White status, education higher than high school, poverty income ratio above 3.5, light activity, BUN, and eGFR.

**Table 1:** Patient characteristics associated with the occurrence of type 2 diabetes mellitus.

Variables	Total	Is there T2DM?		*P* value
		NO	YES	
**Basic Characteristics of the Population**				
**Age, mean (95% CI), years**	59.1 (58.8,59.4)	58.4 (58.1,58.7)	61.8 (61.5,62.2)	<0.001
**Gender, n % (95% CI)**				<0.001
**Male**	47.9 (47.0,48.9)	46.6 (45.5,47.8)	53.1 (50.8,55.4)	
**Female**	52.1 (51.1,53.0)	53.4 (52.2,54.5)	46.9 (44.6,49.2)	
**Race, n % (95% CI)**				<0.001
**Mexican American**	6.2 (5.1,7.6)	5.3 (4.4,6.5)	10.0 (7.9,12.5)	
**Non-Hispanic White**	71.7 (68.9,74.3)	74.7 (72.1,77.1)	59.5 (55.5,63.4)	
**Non-Hispanic Black**	10.3 (8.9,11.8)	9.1 (7.9,10.5)	15.0 (12.9,17.5)	
**Other Race**	11.8 (10.6,13.1)	10.9 (9.8,12.2)	15.4 (13.8,17.3)	
**Education, n % (95% CI)**				<0.001
**Lower than high school**	16.9 (15.5,18.3)	15.0 (13.6,16.5)	24.3 (22.3,26.4)	
**High school diploma**	24.2 (23.0,25.5)	23.8 (22.4,25.3)	25.7 (23.6,27.9)	
**Higher than high school**	58.9 (56.8,61.0)	61.1 (58.8,63.4)	50.0 (47.5,52.5)	
**Poverty income ratio, n % (95% CI)**				<0.001
**<1.3**	16.2 (14.8,17.6)	14.4 (13.0,16.0)	23.2 (21.2,25.3)	
**1.3–3.5**	31.2 (29.7,32.8)	30.2 (28.5,32.0)	35.3 (33.3,37.4)	
**>3.5**	45.0 (42.7,47.3)	47.8 (45.3,50.2)	33.7 (31.1,36.5)	
**Missing**	7.6 (6.9,8.4)	7.6 (6.8,8.4)	7.7 (6.7,8.9)	
**Lifestyle**				
**Thiamine intake, mean (95% CI), mg**	1.6 (1.6,1.6)	1.6 (1.6,1.6)	1.5 (1.5,1.6)	0.006
**Smoking status, n % (95% CI)**				<0.001
**Never**	52.6 (51.3,53.9)	53.5 (52.0,55.0)	49.1 (46.7,51.4)	
**Former**	30.6 (29.5,31.7)	29.2 (28.0,30.6)	35.9 (33.6,38.2)	
**Current**	16.8 (15.9,17.8)	17.3 (16.1,18.5)	15.1 (13.6,16.7)	
**Alcohol use, n % (95% CI)**				<0.001
**Never**	9.4 (8.6,10.3)	8.7 (7.8,9.6)	12.3 (11.0,13.7)	
**Moderate**	41.7 (40.3,43.2)	42.3 (40.8,43.9)	39.2 (36.8,41.7)	
**Heavy**	34.0 (32.7,35.4)	35.3 (33.8,36.8)	28.8 (26.4,31.2)	
**Missing**	14.9 (14.0,15.9)	13.7 (12.7,14.8)	19.7 (18.2,21.4)	
**Physical activity, n % (95% CI)**				<0.001
**vigorous activity**	31.4 (30.0,32.7)	28.4 (27.0,29.9)	43.2 (40.9,45.6)	
**moderate activity**	35.7 (34.4,36.9)	35.7 (34.4,37.0)	35.7 (33.4,38.1)	
**light activity**	33.0 (31.6,34.4)	35.9 (34.4,37.5)	21.1 (19.3,23.0)	
**Health checkup**				
**BMI, mean (95% CI), kg/m2**				<0.001
**< 25.0 kg/m2**	24.7 (23.6,25.9)	28.2 (27.0,29.6)	10.4 (9.1,12.0)	
**25.0–29.9 kg/m2**	35.4 (34.2,36.5)	37.5 (36.2,38.8)	26.8 (24.7,28.9)	
**>29.9 kg/m2**	39.9 (38.5,41.3)	34.3 (32.9,35.7)	62.8 (60.1,65.4)	
**WC, mean (95% CI), cm**	101.9 (101.4,102.4)	99.6 (99.1,100.1)	111.0 (110.2,111.8)	<0.001
**Laboratories**				
**Total calcium, mean (95% CI), mg/dL**	9.4 (9.4,9.4)	9.4 (9.4,9.4)	9.4 (9.4,9.4)	0.001
**BUN, mean (95% CI), mg/dL**	14.9 (14.8,15.1)	14.5 (14.4,14.7)	16.5 (16.2,16.8)	<0.001
**TBIL, mean (95% CI), mg/dL**	0.7 (0.6,0.7)	0.7 (0.7,0.7)	0.6 (0.6,0.6)	0.001
**SUA, mean (95% CI), mg/dL**	5.5 (5.5,5.5)	5.4 (5.4,5.5)	5.7 (5.7,5.8)	<0.001
**TG, mean (95% CI), mg/dL**	160.9 (157.8,164.0)	152.0 (148.8,155.2)	197.0 (188.7,205.2)	<0.001
**TC, mean (95% CI), mg/dL**	199.5 (198.4,200.6)	203.7 (202.6,204.9)	182.3 (180.1,184.6)	<0.001
**HDL-C, mean (95% CI), mg/dL**	54.7 (54.1,55.3)	56.5 (55.9,57.1)	47.3 (46.5,48.1)	<0.001
**eGFR, mean (95% CI), ml/min/1.73m** ^**2**^	81.3 (80.7,81.9)	81.4 (80.6,82.1)	81.1 (80.1,82.1)	0.647
**UACR, mean (95% CI), mg/g**	38.7 (35.0,42.5)	20.5 (18.1,23.0)	112.3 (94.1,130.4)	<0.001
**Disease diagnosis**				
**Hypertension, n % (95% CI)**	58.4 (57.0,59.7)	53.9 (52.4,55.4)	76.5 (74.3,78.5)	<0.001
**CHF, n % (95% CI)**	3.3 (2.9,3.6)	2.1 (1.8,2.4)	7.9 (6.8,9.2)	<0.001
**CHD, n % (95% CI)**	5.5 (4.9,6.1)	4.1 (3.5,4.7)	11.1 (9.8,12.6)	<0.001
**Angina, n % (95% CI)**	3.2 (2.8,3.7)	2.2 (1.8,2.6)	7.5 (6.3,8.9)	<0.001
**Stroke, n % (95% CI)**	4.1 (3.8,4.4)	3.2 (2.9,3.6)	7.6 (6.6,8.8)	<0.001
**Heart attack, n % (95% CI)**	5.0 (4.5,5.5)	3.6 (3.1,4.0)	10.7 (9.4,12.1)	<0.001
**CVD, n % (95% CI)**	12.5 (11.8,13.3)	9.6 (8.9,10.3)	24.5 (22.6,26.5)	<0.001

**Table 2:** Characteristics of the population describing dietary thiamine intake.

Variables	Total	Dietary Thiamine intake (mg) (log2 transform) (quartile)				p value
		Q1	Q2	Q3	Q4	
**Basic Characteristics of the Population**						
**Age, mean (95% CI), years**	59.1 (58.8,59.4)	60.0 (59.6,60.5)	59.9 (59.4,60.4)	59.2 (58.7,59.6)	57.6 (57.2,58.0)	<0.001
**Gender, n % (95% CI)**						<0.001
**Male**	47.9 (47.0,48.9)	27.1 (25.4,28.9)	38.7 (36.4,41.1)	48.9 (47.0,50.9)	70.6 (68.4,72.6)	
**Female**	52.1 (51.1,53.0)	72.9 (71.1,74.6)	61.3 (58.9,63.6)	51.1 (49.1,53.0)	29.4 (27.4,31.6)	
**Race, n % (95% CI)**						<0.001
**Mexican American**	6.2 (5.1,7.6)	7.0 (5.7,8.7)	6.6 (5.3,8.1)	5.6 (4.3,7.2)	5.9 (4.8,7.4)	
**Non-Hispanic White**	71.7 (68.9,74.3)	63.7 (60.0,67.2)	70.9 (67.5,74.1)	74.4 (71.4,77.1)	75.8 (73.1,78.3)	
**Non-Hispanic Black**	10.3 (8.9,11.8)	16.3 (14.0,19.0)	10.6 (9.0,12.4)	8.7 (7.4,10.1)	7.0 (6.0,8.1)	
**Other Race**	11.8 (10.6,13.1)	12.9 (11.3,14.8)	11.9 (10.3,13.8)	11.4 (10.0,12.9)	11.3 (9.8,12.9)	
**Education, n % (95% CI)**						<0.001
**Lower than high school**	16.9 (15.5,18.3)	22.1 (20.2,24.1)	18.3 (16.3,20.5)	14.6 (12.9,16.6)	13.7 (12.3,15.3)	
**High school diploma**	24.2 (23.0,25.5)	25.1 (23.1,27.2)	24.2 (22.3,26.2)	24.9 (22.9,27.0)	22.9 (20.8,25.3)	
**Higher than high school**	58.9 (56.8,61.0)	52.8 (49.8,55.8)	57.5 (54.6,60.4)	60.5 (57.9,63.1)	63.3 (60.6,66.0)	
**Poverty income ratio, n % (95% CI)**						<0.001
**<1.3**	16.2 (14.8,17.6)	21.6 (19.7,23.6)	16.3 (14.4,18.5)	14.3 (12.7,16.1)	13.6 (12.0,15.4)	
**1.3–3.5**	31.2 (29.7,32.8)	33.8 (31.7,36.0)	32.8 (30.6,35.1)	31.0 (28.6,33.6)	28.1 (25.6,30.8)	
**>3.5**	45.0 (42.7,47.3)	35.7 (32.7,38.7)	43.5 (40.4,46.6)	46.5 (43.5,49.4)	52.0 (49.1,54.9)	
**Missing**	7.6 (6.9,8.4)	8.9 (7.8,10.2)	7.4 (6.4,8.7)	8.2 (7.1,9.5)	6.3 (5.3,7.5)	
**Lifestyle**						
**Smoking status, n % (95% CI)**						<0.001
**Never**	52.6 (51.3,53.9)	52.9 (50.6,55.1)	54.0 (51.6,56.4)	53.1 (50.7,55.6)	50.8 (48.5,53.0)	
**Former**	30.6 (29.5,31.7)	27.5 (25.4,29.7)	30.0 (27.6,32.4)	32.0 (29.9,34.2)	32.0 (30.0,34.1)	
**Current**	16.8 (15.9,17.8)	19.6 (17.8,21.5)	16.1 (14.4,17.9)	14.9 (13.1,16.7)	17.2 (15.7,18.8)	
**Alcohol use, n % (95% CI)**						<0.001
**Never**	9.4 (8.6,10.3)	11.6 (10.2,13.1)	10.1 (8.7,11.7)	8.9 (7.8,10.2)	7.6 (6.3,9.1)	
**Moderate**	41.7 (40.3,43.2)	36.1 (33.9,38.4)	41.0 (38.5,43.6)	44.4 (41.9,47.0)	44.0 (41.5,46.6)	
**Heavy**	34.0 (32.7,35.4)	36.3 (33.8,38.8)	33.7 (30.9,36.6)	31.5 (29.5,33.6)	34.8 (32.5,37.2)	
**Missing**	14.9 (14.0,15.9)	16.0 (14.4,17.8)	15.2 (13.6,17.0)	15.1 (13.6,16.8)	13.5 (12.2,15.0)	
**Physical activity, n % (95% CI)**						<0.001
**vigorous activity**	31.4 (30.0,32.7)	38.9 (36.8,41.1)	32.8 (30.6,35.1)	29.7 (27.7,31.7)	26.0 (23.9,28.2)	
**moderate activity**	35.7 (34.4,36.9)	34.2 (32.3,36.2)	36.8 (34.4,39.3)	39.6 (37.2,42.0)	32.2 (29.8,34.6)	
**light activity**	33.0 (31.6,34.4)	26.9 (24.9,29.0)	30.4 (28.1,32.7)	30.8 (28.4,33.2)	41.8 (39.1,44.6)	
**Health checkup**						
**BMI, mean (95% CI), kg/m** ^**2**^						0.153
**< 25.0 kg/m** ^**2**^	24.7 (23.6,25.9)	25.5 (23.4,27.6)	24.7 (22.7,26.9)	24.7 (22.4,27.1)	24.2 (22.4,26.1)	
**25.0–29.9 kg/m** ^**2**^	35.4 (34.2,36.5)	33.9 (31.6,36.3)	33.4 (31.1,35.7)	36.1 (34.0,38.3)	37.4 (35.3,39.7)	
**>29.9 kg/m** ^**2**^	39.9 (38.5,41.3)	40.6 (38.4,43.0)	41.9 (39.4,44.4)	39.2 (36.7,41.8)	38.4 (36.1,40.7)	
**WC, mean (95% CI), cm**	101.9 (101.4,102.4)	100.4 (99.6,101.2)	102.1 (101.2,102.9)	101.7 (100.8,102.5)	103.0 (102.2,103.8)	<0.001
**Laboratories**						
**Total calcium, mean (95% CI), mg/dL**	9.4 (9.4,9.4)	9.4 (9.4,9.4)	9.4 (9.4,9.4)	9.4 (9.4,9.4)	9.4 (9.3,9.4)	<0.001
**BUN, mean (95% CI), mg/dL**	14.9 (14.8,15.1)	14.5 (14.2,14.8)	14.9 (14.6,15.2)	15.0 (14.8,15.3)	15.2 (14.9,15.4)	<0.001
**TBIL, mean (95% CI), mg/dL**	0.7 (0.6,0.7)	0.6 (0.6,0.6)	0.6 (0.6,0.7)	0.7 (0.7,0.7)	0.7 (0.7,0.7)	<0.001
**SUA, mean (95% CI), mg/dL**	5.5 (5.5,5.5)	5.4 (5.4,5.5)	5.5 (5.4,5.5)	5.4 (5.4,5.5)	5.6 (5.6,5.7)	<0.001
**TG, mean (95% CI), mg/dL**	160.9 (157.8,164.0)	146.2 (142.4,150.1)	162.1 (156.4,167.8)	160.2 (154.5,165.8)	171.6 (165.6,177.7)	<0.001
**TC, mean (95% CI), mg/dL**	199.5 (198.4,200.6)	204.1 (202.1,206.1)	201.3 (199.4,203.2)	198.0 (195.7,200.3)	195.9 (194.0,197.9)	<0.001
**HDL-C, mean (95% CI), mg/dL**	54.7 (54.1,55.3)	58.4 (57.4,59.5)	55.1 (54.3,55.9)	54.4 (53.5,55.3)	51.8 (51.0,52.7)	<0.001
**eGFR, mean (95% CI), ml/min/1.73m** ^**2**^	81.3 (80.7,81.9)	80.2 (79.1,81.4)	80.8 (79.9,81.8)	81.2 (80.3,82.0)	82.6 (81.7,83.6)	0.003
**UACR, mean (95% CI), mg/g**	38.7 (35.0,42.5)	39.6 (33.7,45.6)	41.1 (32.0,50.2)	40.7 (31.5,50.0)	34.2 (26.1,42.3)	0.657
**Disease diagnosis**						
**Hypertension, n % (95% CI)**	58.4 (57.0,59.7)	61.5 (58.9,63.9)	59.2 (56.8,61.5)	56.3 (53.9,58.6)	57.3 (54.6,60.0)	0.018
**CHF, n % (95% CI)**	3.3 (2.9,3.6)	3.9 (3.3,4.6)	3.8 (3.2,4.6)	3.2 (2.6,3.8)	2.4 (1.8,3.1)	0.003
**CHD, n % (95% CI)**	5.5 (4.9,6.1)	4.9 (4.1,5.7)	5.9 (4.8,7.2)	5.4 (4.5,6.5)	5.6 (4.7,6.7)	0.447
**Angina, n % (95% CI)**	3.2 (2.8,3.7)	3.5 (2.7, 4.5)	3.2 (2.6,3.9)	2.6 (2.1,3.3)	3.6 (2.9,4.6)	0.208
**Stroke, n % (95% CI)**	4.1 (3.8,4.4)	5.5 (4.7,6.4)	4.2 (3.5,5.1)	3.6 (2.9,4.4)	3.4 (2.8,4.1)	<0.001
**Heart attack, n % (95% CI)**	5.0 (4.5,5.5)	5.2 (4.4,6.1)	4.9 (4.2,5.7)	5.0 (4.2,6.0)	4.8 (4.0,5.7)	0.910
**CVD, n % (95% CI)**	12.5 (11.8,13.3)	13.8 (12.4, 15.3)	12.9 (11.5,14.5)	12.2 (10.9,13.5)	11.6 (10.4,12.9)	0.122
**T2DM, n % (95% CI)**	19.8 (18.9,20.8)	21.5 (19.7,23.4)	20.3 (18.5,22.2)	19.9 (18.2,21.8)	18.1 (16.7,19.6)	0.038

### Univariate analysis of T2DM

Univariate analysis (Table 3) revealed that dietary thiamine intake, age, gender, race, education, poverty income ratio, alcohol use, smoking status, physical activity, BUN, total calcium, TBIL, SUA, HDL-C, TC, WC, BMI, hypertension, CVD, CHF, CHD, angina, stroke, and heart attack were all factors associated with a higher incidence of T2DM. Furthermore, neither TG nor eGFR nor UACR showed significant associations with T2DM risk. Of these significant correlations, non-Hispanic whites and other races were each negatively correlated compared to Mexican Americans (OR = 0.42, 95% CI: 0.37, 0.49; OR = 0.75, 95% CI: 0.65, 0.88). Compared with those who completed lower than high school, those who completed high school and those who completed higher education were negatively correlated (OR = 0.67, 95% CI: 0.58, 0.77; OR = 0.51, 95% CI: 0.45, 0.57). Compared with low-income levels, middle-income and higher-income levels were negatively correlated (OR = 0.73, 95% CI: 0.63, 0.83; OR = 0.44, 95% CI: 0.38, 0.51). Moderate alcohol use and heavy alcohol use were each negatively correlated compared to never alcohol use (OR = 0.65, 95% CI: 0.56, 0.76; OR = 0.57, 95% CI: 0.49, 0.68). Compared with never smokers, former smokers were more positively correlated (OR = 1.34, 95% CI: 1.18, 1.52). Compared with vigorous physical activity, moderate physical activity and light physical activity were negatively correlated (OR = 0.66, 95% CI: 0.58, 0.74; OR = 0.39, 95% CI: 0.34, 0.44). Compared with a normal weight, overweight and obesity were positively correlated with T2DM (OR = 1.93, 95% CI: 1.64, 2.27; OR = 4.96, 95% CI: 4.21, 5.83). Hypertension, CVD, CHF, CHD, angina, stroke, and heart attack were all positively associated with the risk of T2DM compared to the healthy group (OR = 2.78, 95% CI: 2.46, 3.15; OR = 3.07, 95% CI: 2.71, 3.48; OR = 3.98, 95% CI: 3.22, 4.93; OR = 2.94, 95% CI: 2.50, 3.46; OR = 3.66, 95% CI: 2.83, 4.75; OR = 2.50, 95% CI: 2.04, 3.06; OR = 3.25, 95% CI: 2.71, 3.89). In addition, age, BUN, total calcium, SUA, and WC were positively associated with the risk of T2DM, while dietary thiamine intake, TBIL, HDL-C, and TC were negatively associated with T2DM.

**Table 3:** Univariate analysis of factors associated with type 2 diabetes mellitus, weighted.

Variable	T2DM	
	N	OR (95%CI)
**Basic Characteristics of the Population**		
**Age**	15231	1.03 (1.03, 1.04)
**Gender**		
**Male**	7537	Ref.
**Female**	7694	0.77 (0.69, 0.86)
**Race**		
**Mexican American**	2186	Ref.
**Non-Hispanic White**	6237	0.42 (0.37, 0.49)
**Non-Hispanic Black**	3480	0.88 (0.76, 1.01)
**Other Race**	3328	0.75 (0.65, 0.88)
**Education**		
**Lower than high school**	4106	Ref.
**High school**	3569	0.67 (0.58, 0.77)
**Higher than high school**	7556	0.51 (0.45, 0.57)
**Poverty income ratio**		
**<1.3**	4010	Ref.
**1.3–3.5**	5149	0.73 (0.63, 0.83)
**>3.5**	4536	0.44 (0.38, 0.51)
**Missing data**	1536	0.63 (0.51, 0.78)
**Lifestyle**		
**Dietary thiamine**	15231	0.90 (0.84, 0.97)
**Smoking status**		
**Never**	7962	Ref.
**Former**	4534	1.34 (1.18, 1.52)
**Current**	2735	0.95 (0.82, 1.11)
**Alcohol use**		
**Never**	1740	Ref.
**Moderate**	5718	0.65 (0.56, 0.76)
**Heavy**	4838	0.57 (0.49, 0.68)
**Missing**	2935	1.02 (0.86, 1.20)
**Physical activity**		
**vigorous activity**	5851	Ref.
**moderate activity**	5100	0.66 (0.58, 0.74)
**light activity**	4280	0.39 (0.34, 0.44)
**Health checkup**		
**BMI**		
**< 25.0 kg/m2**	3582	Ref.
**25.0–29.9 kg/m2**	5343	1.93 (1.64, 2.27)
**>29.9 kg/m2**	6306	4.96 (4.21, 5.83)
**WC**	15231	1.05 (1.05, 1.05)
**Laboratories**		
**Total calcium**	15231	1.29 (1.11, 1.49)
**BUN**	15231	1.06 (1.05, 1.07)
**TBIL**	15231	0.67 (0.55, 0.82)
**SUA**	15231	1.17 (1.12, 1.21)
**TG**	15231	1.00 (1.00, 1.00)
**TC**	15231	0.99 (0.98, 0.99)
**HDL-C**	15231	0.96 (0.96, 0.97)
**eGFR**	15231	1.00 (1.00, 1.00)
**UACR**	15231	1.00 (1.00, 1.00)
**Disease diagnosis**		
**Hypertension**		
**No**	5507	Ref.
**Yes**	9724	2.78 (2.46, 3.15)
**CHF**		
**No**	14560	Ref.
**Yes**	671	3.98 (3.22, 4.93)
**CHD**		
**No**	14316	Ref.
**Yes**	915	2.94 (2.50, 3.46)
**Angina**		
**No**	14689	Ref.
**Yes**	542	3.66 (2.83, 4.75)
**Stroke**		
**No**	14411	Ref.
**Yes**	820	2.50 (2.04, 3.06)
**Heart attack**		
**No**	14301	Ref.
**Yes**	930	3.25 (2.71, 3.89)
**CVD**		
**No**	12895	Ref.
**Yes**	2336	3.07 (2.71, 3.48)

### Correlation between dietary thiamine and T2DM

**Fig 2:**
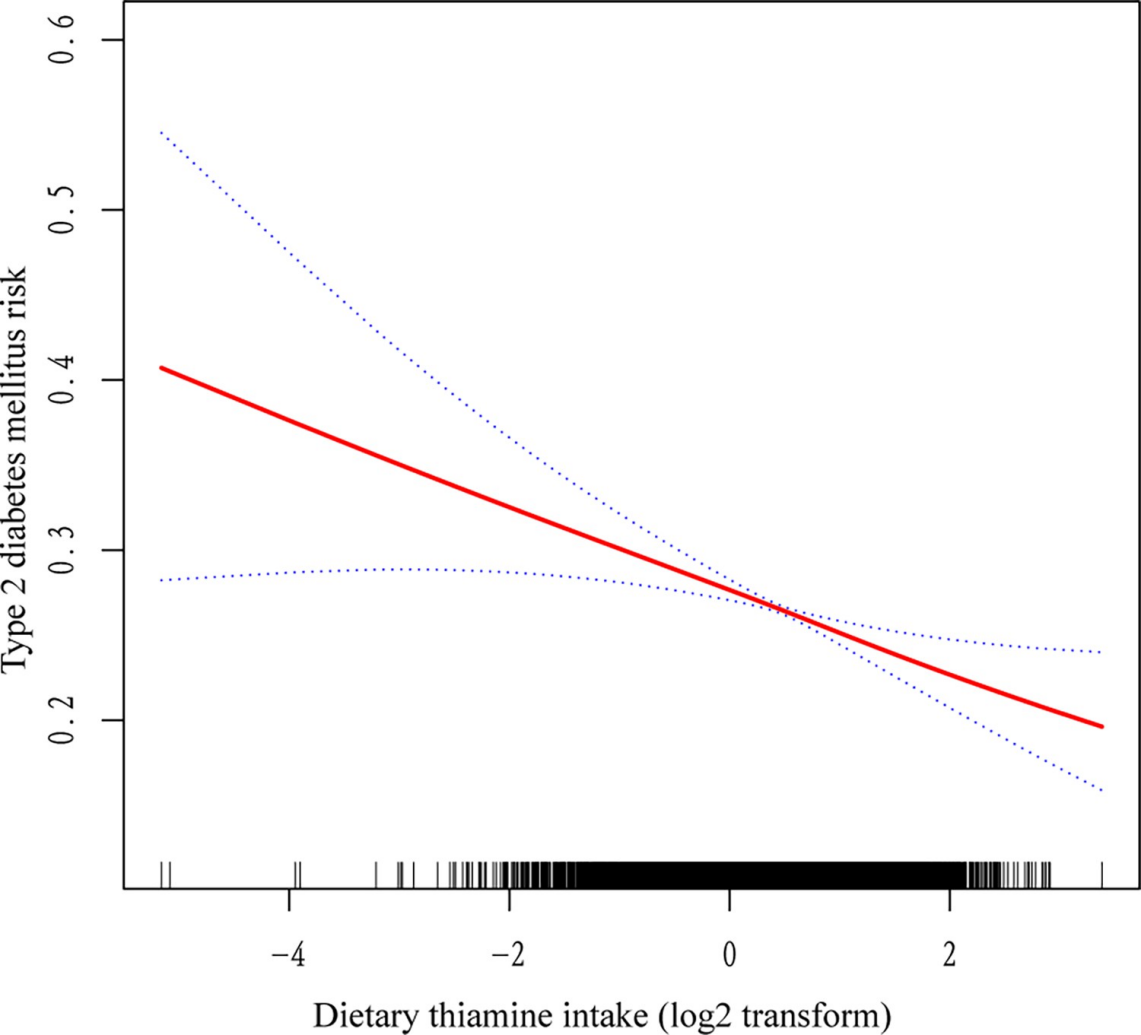
Relationship between dietary thiamine and risk of type 2 diabetes mellitus. The area between the two blue curves represents the 95% CI. The data were adjusted for age, gender, race, education, poverty income ratio, smoking status, alcohol use, physical activity, BMI, WC, total calcium, BUN, TBIL, SUA, TG, TC, HDL-C, eGFR, UACR, hypertension, and CVD.

Based on weighted logistic regression analysis, we examined the correlation between dietary thiamine and T2DM (Table 4). Model 1 (unadjusted model) showed that dietary thiamine intake was negatively associated with T2DM (OR = 0.88, 95% CI: 0.82, 0.95); that is, the risk of T2DM decreased by 12% for each unit increase (log2 transform) in dietary thiamine. Model 2 (microadjusted model) showed that dietary thiamine intake was negatively associated with T2DM (OR = 0.91, 95% CI: 0.84, 0.98); that is, the risk of T2DM decreased by 9% for each unit (log2 transform) increase in dietary thiamine. Model 3 (fully adjusted model) showed that dietary thiamine intake was negatively associated with T2DM (OR = 0.86, 95% CI: 0.78, 0.95); that is, the risk of T2DM decreased by 14% for each unit (log2 transform) increase in dietary thiamine. Sensitivity analyses were conducted based on the quartile groups of dietary thiamine intake (Q1, Q2, Q3, and Q4) to assess the stability of the results. In Model 1, Q2, Q3, and Q4 were all negatively correlated with the risk of T2DM compared to Q1 (OR = 0.93, 95% CI: 0.79, 1.09; OR = 0.91, 95% CI: 0.80, 1.03; OR = 0.80, 95% CI: 0.70, 0.93). In Model 2, Q2, Q3, and Q4 were all negatively correlated with the risk of T2DM compared to Q1 (OR = 0.95, 95% CI: 0.81, 1.13; OR = 0.95, 95% CI: 0.84, 1.08; OR = 0.84, 95% CI: 0.72, 0.98). In Model 3, Q2, Q3, and Q4 were all negatively correlated with the risk of T2DM compared to Q1 (OR = 0.88, 95% CI: 0.74, 1.05; OR = 0.90, 95% CI: 0.76, 1.06; OR = 0.75, 95% CI: 0.62, 0.90). Overall risk trends between the lowest quartile (Q1) and the highest quartile (Q4) of all models were consistent (*p* for trend = 0.003, 0.029, 0.005). Furthermore, a smooth curve fit was plotted, revealing a linear correlation between dietary thiamine intake and T2DM (Fig 2).

**Table 4:** Multivariate logistic regression of dietary thiamine and type 2 diabetes mellitus using various weighted models.

Variable	Model 1	Model 2	Model 3
	OR (95%CI)	OR (95%CI)	OR (95%CI)
**Dietary thiamine intake (mg) (log2 transform)**	0.88 (0.82, 0.95)	0.91 (0.84, 0.98)	0.86 (0.78, 0.95)
**Dietary thiamine intake (mg) (log2 transform) (quartile)**			
**Q1**	Ref.	Ref.	Ref.
**Q2**	0.93 (0.79, 1.09)	0.95 (0.81, 1.13)	0.88 (0.74, 1.05)
**Q3**	0.91 (0.80, 1.03)	0.95 (0.84, 1.08)	0.90 (0.76, 1.06)
**Q4**	0.80 (0.70, 0.93)	0.84 (0.72, 0.98)	0.75 (0.62, 0.90)
***p* for trend**	0.003	0.029	0.005

Model 1: Unadjusted.

Model 2: Adjusted for age, gender, and race.

Model 3: Adjusted for age, gender, race, education, poverty income ratio, smoking status, alcohol use, physical activity, BMI, WC, total calcium, BUN, TBIL, SUA, TG, TC, HDL-C, eGFR, UACR, hypertension, and CVD.

### Subgroup analysis of the correlation between dietary thiamine and T2DM

**Fig 3:**
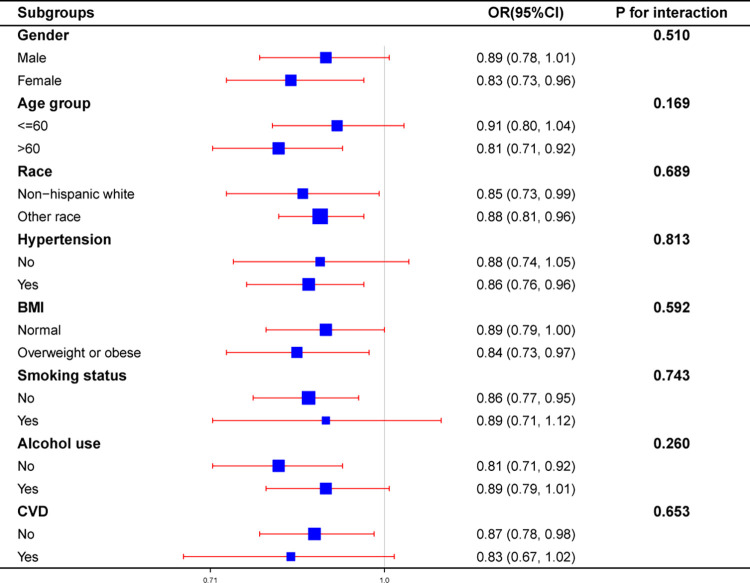
Forest plot of dietary thiamine and type 2 diabetes mellitus. After adjusting for age, gender, race, education, poverty income ratio, smoking status, alcohol use, physical activity, BMI, WC, total calcium, BUN, TBIL, SUA, TG, TC, HDL-C, eGFR, UACR, hypertension, and CVD, stratified variables were not adjusted.

To further determine whether gender, age, race, hypertension, BMI, smoking status, alcohol use, or CVD altered the association between dietary thiamine and T2DM, we performed subgroup analyses (Fig 3). The results showed that the correlation between dietary thiamine and T2DM remained stable in the subgroups stratified by gender, age, race, hypertension, BMI, smoking status, alcohol use, and CVD, with no interaction (*p* for interaction = 0.510, 0.169, 0.689, 0.813, 0.592, 0.743, 0.260, 0.653).

## Discussion

Our study offers significant insights into the potential correlation between dietary thiamine intake and the onset of T2DM among middle-aged and older adults in the US. First, our study revealed a potential negative association between thiamine intake and T2DM risk, regardless of confounding factors. Nevertheless, our data do not establish a causal relationship. This finding may have important clinical and preventive implications.

Initially, our analysis verified that increased thiamine intake through diet is linked to a decreased risk of developing T2DM. This observation concurs with prior research showing that diabetic patients have lower plasma thiamine levels [28]. Similarly, a cross-sectional study in Korea revealed that thiamine intake was negatively associated with the risk of T2DM, especially among people aged 70 years and older [17]. Additionally, a randomized double-blind trial revealed that thiamine supplementation effectively lowered 2-h plasma glucose levels, decreased fasting glucose, and boosted insulin functionality [19]. Notably, a longitudinal study conducted in China revealed a U-shaped association between dietary thiamine intake and the incidence of newly diagnosed T2DM. This finding suggests an optimal thiamine intake range of 0.75 to 1.10 mg/day for minimizing the risk of T2DM [20]. In contrast, a prospective study in Japan revealed no correlation between dietary thiamine intake and T2DM risk [21].

Such discrepancies may be attributed to differences in population characteristics, dietary patterns, or uncontrolled confounders, emphasizing the need for further investigation in this field. Furthermore, our stratified analysis consistently revealed a negative correlation between dietary thiamine intake and T2DM risk across various subgroups, encompassing age, gender, race, smoking status, alcohol use, hypertension, BMI, and CVD. This finding underscores the robustness and generality of our results, indicating broad applicability.

The potential mechanisms underlying this negative association are multifaceted. Thiamine plays a crucial role in carbohydrate metabolism, particularly in the citric acid cycle and pentose phosphate pathway, which are vital for adenosine triphosphate (ATP) generation and nicotinamide adenine dinucleotide phosphate (NADPH) production, respectively [11,29]. NADPH, in turn, is essential for maintaining the redox state of cells and protecting against oxidative stress, a known contributor to diabetes pathogenesis [30–32]. Additionally, the role of thiamine in nerve transmission and neuromuscular function could indirectly influence insulin secretion and sensitivity [33]. In conclusion, our study provides compelling evidence of a negative correlation between dietary thiamine intake and the risk of T2DM among middle-aged and older adults in the US. Although the precise underlying mechanisms are yet to be clarified, the various biochemical roles of thiamine offer credible hypotheses for its defensive benefits. Future investigations should focus on verifying these results across different demographics and elucidating the specific biochemical processes at play.

The NHANES database serves as a strong representation of the US population 45 years and older, constituting a significant strength of this study. Furthermore, our rigorous inclusion criteria and adjustments for potential confounders enhance the reliability of our findings. Nonetheless, our study has its limitations. Primarily, as a cross-sectional study, this study precludes the determination of a causal relationship between dietary thiamine and T2DM. Second, the study excluded individuals under 45 years of age, and some participant data were incomplete. Hence, the applicability of these results to this younger demographic population remains uncertain. Additionally, the intricacies of potential confounders could influence the results. Furthermore, recollection bias could impact self-report surveys that infer thiamine consumption and T2DM diagnosis. These caveats highlight the need for additional prospective studies to prove a causal relationship between thiamine in the diet and T2DM.

## Conclusion

Our study suggested a possible link between dietary thiamine intake and the risk of T2DM in US adults aged 45 years and older. The observed inverse relationship between thiamine intake and T2DM incidence implies substantial clinical and preventative significance. These results pave the way for exploring innovative nutritional strategies to prevent T2DM. However, the specific biological pathways connecting dietary thiamine to T2DM risk need further clarification through future research.

## References

[1] Magliano DJ, Islam RM, Barr E, Gregg EW, Pavkov ME, Harding JL, et al. Trends in incidence of total or type 2 diabetes: systematic review. BMJ. 2019; 366: l5003. doi: 10.1136/bmj.l5003 31511236 PMC6737490

[2] Chan J, Lim LL, Wareham NJ, Shaw JE, Orchard TJ, Zhang P, et al. The Lancet Commission on diabetes: using data to transform diabetes care and patient lives. Lancet. 2021;396(10267):2019–82. doi: 10.1016/S0140-6736(20)32374-6 33189186

[3] Sun H, Saeedi P, Karuranga S, Pinkepank M, Ogurtsova K, Duncan BB, et al. IDF Diabetes Atlas: Global, regional and country-level diabetes prevalence estimates for 2021 and projections for 2045. Diabetes Res Clin Pract. 2022; 183:109119. doi: 10.1016/j.diabres.2021.109119 34879977 PMC11057359

[4] Harding JL, Pavkov ME, Magliano DJ, Shaw JE, Gregg EW. Global trends in diabetes complications: a review of current evidence. Diabetologia. 2019;62(1):3–16. doi: 10.1007/s00125-018-4711-2 30171279

[5] Yuchen C, Hejia Z, Fanke M, Qixin D, Liyang C, Xi G, et al. Exploring the shared molecular mechanism of microvascular and macrovascular complications in diabetes: Seeking the hub of circulatory system injury. Front Endocrinol (Lausanne). 2023; 14:1032015. doi: 10.3389/fendo.2023.1032015 36755923 PMC9899888

[6] Zheng Y, Ley SH, Hu FB. Global aetiology and epidemiology of type 2 diabetes mellitus and its complications. Nat Rev Endocrinol. 2018;14(2):88–98. doi: 10.1038/nrendo.2017.151 29219149

[7] Martín-Peláez S, Fito M, Castaner O. Mediterranean Diet Effects on Type 2 Diabetes Prevention, Disease Progression, and Related Mechanisms. A Review. Nutrients. 2020;12(8). 10.3390/nu12082236. PMC7468821 32726990

[8] Toi PL, Anothaisintawee T, Chaikledkaew U, Briones JR, Reutrakul S, Thakkinstian A. Preventive Role of Diet Interventions and Dietary Factors in Type 2 Diabetes Mellitus: An Umbrella Review. Nutrients. 2020;12(9). 10.3390/nu12092722. PMC7551929 32899917

[9] Sobiecki JG, Imamura F, Davis CR, Sharp SJ, Koulman A, Hodgson JM, et al. A nutritional biomarker score of the Mediterranean diet and incident type 2 diabetes: Integrated analysis of data from the MedLey randomised controlled trial and the EPIC-InterAct case-cohort study. Plos Med. 2023;20(4): e1004221. doi: 10.1371/journal.pmed.1004221 37104291 PMC10138823

[10] Maroto-Rodriguez J, Ortolá R, Carballo-Casla A, Iriarte-Campo V, Salinero-Fort MÁ, Rodríguez-Artalejo F, et al. Association between a mediterranean lifestyle and Type 2 diabetes incidence: a prospective UK biobank study. Cardiovasc Diabetol. 2023;22(1):271. doi: 10.1186/s12933-023-01999-x 37794451 PMC10552305

[11] Strobbe S, Verstraete J, Stove C, Van Der Straeten D. Metabolic engineering provides insight into the regulation of thiamin biosynthesis in plants. Plant Physiol. 2021;186(4):1832–47. doi: 10.1093/plphys/kiab198 33944954 PMC8331165

[12] Kerns JC, Arundel C, Chawla LS. Thiamin deficiency in people with obesity. Adv Nutr. 2015;6(2):147–53. doi: 10.3945/an.114.007526 25770253 PMC4352173

[13] Marrs C, Lonsdale D. Hiding in Plain Sight: Modern Thiamine Deficiency. Cells-Basel. 2021;10(10). doi: 10.3390/cells10102595 34685573 PMC8533683

[14] Smith TJ, Johnson CR, Koshy R, Hess SY, Qureshi UA, Mynak ML, et al. Thiamine deficiency disorders: a clinical perspective. Ann N Y Acad Sci. 2021;1498(1):9–28. doi: 10.1111/nyas.14536 33305487 PMC8451766

[15] Sinha S, Kataria A, Kolla BP, Thusius N, Loukianova LL. Wernicke Encephalopathy-Clinical Pearls. Mayo Clin Proc. 2019;94(6):1065–72. doi: 10.1016/j.mayocp.2019.02.018 31171116

[16] Li D, Guo Y, Xia M, Zhang J, Zang W. Dietary intake of thiamine and riboflavin in relation to severe headache or migraine: A cross-sectional survey. Headache. 2022;62(9):1133–42. doi: 10.1111/head.14384 36047917

[17] Duc HN, Oh H, Yoon IM, Kim MS. Association between levels of thiamine intake, diabetes, cardiovascular diseases and depression in Korea: a national cross-sectional study. J Nutr Sci. 2021;10: e31. doi: 10.1017/jns.2021.23 34094512 PMC8141681

[18] Ge Y, Huang S, Li Y, Zhang Z, Kong M, Cui N, et al. Pregnancy thiamine and riboflavin intake and the risk of gestational diabetes mellitus: A prospective cohort study. Am J Clin Nutr. 2023;117(2):426–35. doi: 10.1016/j.ajcnut.2022.11.014 36811572

[19] Alaei SF, Soares MJ, Zhao Y, Sherriff J. High-dose thiamine supplementation improves glucose tolerance in hyperglycemic individuals: A randomized, double-blind cross-over trial. Eur J Nutr. 2013;52(7):1821–4. doi: 10.1007/s00394-013-0534-6 23715873

[20] Liu C, Meng Q, Zu C, Li R, Yang S, He P, et al. U-shaped association between dietary thiamine intake and new-onset diabetes: A nationwide cohort study. QJM. 2022;115(12):822–9. doi: 10.1093/qjmed/hcac159 35894803 PMC9744247

[21] Eshak ES, Iso H, Muraki I, Tamakoshi A. Among the water-soluble vitamins, dietary intakes of vitamins C, B2 and folate are associated with the reduced risk of diabetes in Japanese women but not men. Br J Nutr. 2019;121(12):1357–64. doi: 10.1017/S000711451900062X 30890201

[22] 2. Classification and Diagnosis of Diabetes: Standards of Medical Care in Diabetes-2022. Diabetes Care. 2022;45(Suppl 1): S17–38. 10.2337/dc22-S002. 34964875

[23] Zhang X, Ardeshirrouhanifard S, Li J, Li M, Dai H, Song Y. Associations of Nutritional, Environmental, and Metabolic Biomarkers with Diabetes-Related Mortality in U.S. Adults: The Third National Health and Nutrition Examination Surveys between 1988–1994 and 2016. Nutrients. 2022;14(13). doi: 10.3390/nu14132629 35807807 PMC9268621

[24] Ziegler D, Reiners K, Strom A, Obeid R. Association between diabetes and thiamine status—A systematic review and meta-analysis. Metabolism. 2023; 144:155565. doi: 10.1016/j.metabol.2023.155565 37094704

[25] Whelton PK, Carey RM, Aronow WS, Casey DJ, Collins KJ, Dennison HC, et al. 2017 ACC/AHA/AAPA/ABC/ACPM/AGS/APhA/ASH/ASPC/NMA/PCNA Guideline for the Prevention, Detection, Evaluation, and Management of High Blood Pressure in Adults: Executive Summary: A Report of the American College of Cardiology/American Heart Association Task Force on Clinical Practice Guidelines. Circulation. 2018;138(17): e426–83. doi: 10.1161/CIR.0000000000000597 30354655

[26] Ghaferi AA, Schwartz TA, Pawlik TM. STROBE Reporting Guidelines for Observational Studies. Jama Surg. 2021;156(6):577–8. doi: 10.1001/jamasurg.2021.0528 33825815

[27] von Elm E, Altman DG, Egger M, Pocock SJ, Gøtzsche PC, Vandenbroucke JP. The Strengthening the Reporting of Observational Studies in Epidemiology (STROBE) Statement: guidelines for reporting observational studies. Int J Surg. 2014;12(12):1495–9. doi: 10.1016/j.ijsu.2014.07.013 25046131

[28] Thornalley PJ, Babaei-Jadidi R, Al AH, Rabbani N, Antonysunil A, Larkin J, et al. High prevalence of low plasma thiamine concentration in diabetes linked to a marker of vascular disease. Diabetologia. 2007;50(10):2164–70. doi: 10.1007/s00125-007-0771-4 17676306 PMC1998885

[29] Kennedy DO. B Vitamins and the Brain: Mechanisms, Dose and Efficacy—A Review. Nutrients. 2016;8(2):68. doi: 10.3390/nu8020068 26828517 PMC4772032

[30] DiNicolantonio JJ, Liu J, O’Keefe JH. Thiamine and Cardiovascular Disease: A Literature Review. Prog Cardiovasc Dis. 2018;61(1):27–32. doi: 10.1016/j.pcad.2018.01.009 29360523

[31] Pacei F, Tesone A, Laudi N, Laudi E, Cretti A, Pnini S, et al. The Relevance of Thiamine Evaluation in a Practical Setting. Nutrients. 2020;12(9). 10.3390/nu12092810 PMC7551939 32933220

[32] Beltramo E, Berrone E, Tarallo S, Porta M. Effects of thiamine and benfotiamine on intracellular glucose metabolism and relevance in the prevention of diabetic complications. Acta Diabetol. 2008;45(3):131–41. doi: 10.1007/s00592-008-0042-y 18581039

[33] Rad MG, Sharifi M, Meamar R, Soltani N. The role of pancreas to improve hyperglycemia in STZ-induced diabetic rats by thiamine disulfide. Nutr Diabetes. 2022;12(1):32. doi: 10.1038/s41387-022-00211-5 35725834 PMC9209469

